# Effect of bisphenol F, an analog of bisphenol A, on the reproductive functions of male rats

**DOI:** 10.1186/s12199-019-0797-5

**Published:** 2019-06-10

**Authors:** Asad Ullah, Madeeha Pirzada, Tayyaba Afsar, Suhail Razak, Ali Almajwal, Sarwat Jahan

**Affiliations:** 1 Reproductive Physiology Lab, Department of Animal Sciences, Quaid-i-Azam University, Islamabad, Pakistan; 2 Department of Community Health Sciences, College of Applied Medical Sciences, King Saud University, Riyadh, Kingdom of Saudi Arabia

**Keywords:** Bisphenol F, Male reproductive system, Reproductive toxicity, Antioxidant enzymes, Oxidative stress

## Abstract

**Objective:**

Bisphenol A (BPA) is a monomer primarily used in the production of polycarbonate plastic and epoxy resins. Bisphenol F (BPF) is apparently the main BPA replacement that is used increasingly. BPF has been detected in canned food, thermal paper receipts, and soft drinks. In the present experiment, we did both in vitro and in vivo studies to evaluate the effect of low and high-dose BPF exposures on testosterone concentration, oxidative stress, and antioxidants activity in reproductive tissues of male rats.

**Methods:**

Adult (80–90 days old) male Sprague Dawley rats (*n* = 36) obtained from the rodent colony of Animal Sciences Department of Quaid-i-Azam University. The direct effects of BPF on the antioxidant enzymes and testosterone secretion were measured in vitro and in vivo studies. In an in vivo experiment, adult male Sprague Dawley rats (*n* = 42) were exposed to different concentrations of bisphenol F (1, 5, 25, and 50 mg/kg/d) for 28 days. Various biochemical parameters were analyzed including the level of catalase (CAT), superoxide dismutase (SOD), peroxidase (POD), reactive oxygen species (ROS), and lipid peroxidation (LPO). Moreover, sperm motility, daily sperm production (DSP), comet assay, and histological analysis were performed.

**Results:**

In vitro study showed that BPF exposure significantly (*p* < 0.05) induced oxidative stress biomarkers, i.e., ROS and LPO, while it did not change antioxidant enzyme and testicular testosterone concentration. Whereas, an in vivo study revealed that BPF induced dose-dependent effect and high-dose (100 mg/kg) exposure of BPF significantly reduced tissue protein (*p* < 0.05) content, CAT (*p* < 0.001), SOD (*p* < 0.05), and POD (*p* < 0.05) levels while significantly (*p* < 0.05) augmented ROS and lipid peroxidation. Furthermore, BPF reduces testosterone, LH, and FSH secretion in a dose-dependent manner. Significant (*p* < 0.001) reduction in plasma and intra-testicular testosterone, LH, and FSH was noticed at 100 mg/kg BFP dose. High-dose exposure reduces spermatogenesis.

**Conclusion:**

BPF showed an antagonistic effect on male reproductive hormones and induce alterations in testicular morphology. Increased oxidative stress and decreased testicular antioxidant status might be the underlying mechanism of BFP-induced testicular toxicity.

## Introduction

Bisphenol A (BPA) is a monomer primarily used in the production of polycarbonate plastics and epoxy resins [1, 2]. BPA used in thermal papers does not bind covalently with macromolecules of polymer, and with ease migrates into food and beverages [2]. BPA’s possible route of exposure in humans is food, drinking water, and beverages except for occupational exposure [3]. Studies have shown that beverages in cans are more contaminated with BPA and its analog than those packed in glass containers [2]. Following the restrictions on the use of BPA in the canning industry moving towards safer alternatives of BPA [4], among the BPA alternatives, a large class of compounds shares chemical and physical properties with BPA with variable toxicity and higher estrogenic activities. Among this group of compounds, bisphenol F (BPF) is apparently the main replacement to BPA. BPF has been detected in canned food, thermal paper receipts, and soft drinks. BPF has also exhibited endocrine-modulating capabilities and its toxicity has also shown genotoxic effects, carcinogenic potencies, reproductive complacencies, and oxidative stress [5–8].

BPF has a wide spectrum use in the plastic industry and it has been detected in 55 of the 100 tested urine samples with a concentration of 0.08 μg/L [9]. Similar, concentrations of BPF were observed from 600 urine samples collected in the US from 2000 to 2014 with a concentration of 0.15–0.54 μg/L [10, 11]. HepG2 cell line treated with BPF resulted in oxidative stress and endocrine activities. In another study on HepG2 cells, it was observed that BPF has a higher affinity for ER α and β receptors than that of other bisphenols [12–14]. Significantly less information about potential adverse health outcomes is available about BPF regarding its toxicity. Similar to BPA, BPF is an endocrine-disrupting chemical and displays hormonal activity, with similar average estrogenic, androgenic, and antiestrogen potencies across different in vitro assays. BPF differentially affects signaling pathways involved in lipid metabolism and adipogenesis and causes DNA damage. The present study aimed to examine the possible effects of BPF exposure on the reproductive system of mammals by using rats as an animal model.

## Methods

### Chemicals

Bisphenol F (BPF) with 99% purity was purchased from Santa Cruz Biotechnologies, USA. For the in vitro experiment, different materials as fetal bovine serum, penicillin/streptomycin, and Dulbecco’s modified Eagle’s medium (DMEM) were obtained from Thermos Fisher Scientific (Waltham, MA, USA). CAT, N-acetyl-L-cysteine (NAC) and H_2_O_2_, Ca^2+^, Mg^2+^, Hank’s balanced salt solution (HBSS) were bought from Sigma-Aldrich (St. Louis, MO, USA).

### Animals

Sprague Dawley adult male rats (age 80–90 days) were obtained from the animal facility of Quaid-i-Azam University, Islamabad. Prior to the start of the experiment standard laboratory conditions were maintained. Animals were fed with laboratory feed and tap water was available freely for the animals. Protocols of handling of the animals were approved by the animal sciences department ethical committee.

### Experimental design

For bisphenol F (BPF) exposure on male rats, different experiments were conducted. Firstly, we conducted an in vitro experiment in which the direct effects of BPF on the levels of antioxidant enzymes and different concentrations of testosterone in the testis of rats were tested. While on the results of the in vitro study, an in vivo study was conducted in which the effects of different concentrations of BPF on the reproductive system of male rats were evaluated through subchronic study.

### In vitro studies

In the in vitro study, a total of (*n* = 36 and *n* = 6 animals per group) Sprague Dawley male adult rats were used. In order to investigate the direct effects of BPF on the antioxidant enzymes and testosterone production, an in vitro study was conducted. In this study, different doses of BPF (0, 1, 10, 25, 50, and 100 ng/ml) were prepared in ethanol which was in accordance with [15, 16]. The culturing of testicular tissues was done by the method of [16] with little modifications. Healthy male rats were euthanized and the testes were removed and placed in clean Petri dishes and were cut in equal parts and placed in culture tubes. Culture media containing Dulbecco’s, penicillin, sodium bicarbonate, and streptomycin were mixed with 0, 1, 10, 25, 50, and 100 ng/ml of BPF with the method explained elsewhere by [17]. All the culture tubes containing media, testicular tissues, and BPF different concentrations were incubated in a carbon dioxide (CO_2_) incubator for 2 hours. After the incubation period, all the incubated tissues were washed with saline and homogenized in 30 ml of phosphate buffer saline (PBS) and centrifuged at 30,000 for 30 min. Then the supernatant was collected and stored at − 80 °C for further investigation.

### In vivo study

Adult male Sprague Dawley rats (*n* = 42) were divided into six groups (*n* = 7/group) by randomization procedures explained elsewhere [18]. All the animals were exposed to different concentrations (1, 5, 25, 50, and 100 mg/kg body weight/ day) of BPF for 28 days.

Group 1: Control received saline

Group 2: Administration of BPF at a dose of 1 mg/kg body weight/day

Group 3: Administration of BPF at a dose of 5 mg/kg body weight/day

Group 4: Administration of BPF at a dose of 25 mg/kg body weight/day

Group 5: Administration of BPF at a dose of 50 mg/kg body weight/day

Group 6: Administration of BPF at a dose of 100 mg/kg body weight/day

No mortality was recorded during the period of experimentation. At the end of the experiment (on the 29th day), animals were euthanized and different organs were dissected and stored at − 80 °C for different tests. Blood was collected and centrifuged at 3000 rpm for 10 mins and plasma was separated and stored at − 20 °C for hormonal and different biochemical analysis by the researcher blind to the treatment groups. The reproductive organs as testicular tissues (left testis and left epididymis) were weighed and processed for antioxidant enzymes while right testis (transverse sections) and right epididymis were fixed in 10% formalin for histological analysis as explained by [19].

### Biochemical analysis

Tissues collected from both in vitro and in vivo studies were further processed for the antioxidant enzymes and oxidative stress markers. Tissues were homogenized with an automatic homogenizer in phosphate buffer saline (PBS) and centrifuged at 30,000 rpm for 30 mins. After the centrifugation, the supernatant was removed and used for the hormonal analysis, protein estimation, and antioxidant enzymes [17, 19].

### Catalase (CAT)

Afsar et al.’s method was used to determine the catalase (CAT) activity [20], and the change in the absorbance was measured in the tissues. In this assay 50 ml, the homogenate was diluted in 2 ml of phosphate buffer with a pH of 7.0. After mixing it thoroughly the absorbance was read at 240 nm with an interval of 15 s and 30 s. Change in the absorbance of 0.01 as unit/min was defined as one unit of CAT.

### Superoxide dismutase (SOD)

Afsar and colleagues method was used to determine the superoxide dismutase (SOD) activity [21]. In this assay, the amount of chromogen formed was measured at 560 nm. The results were expressed in units per milligram of protein.

### Peroxidase (POD)

Peroxidase (POD) activity in the homogenate was determined by the spectrophotometric method of Carlberg and Mannervik, [22]. In this assay, the homogenate was mixed with 0.1 ml of guaiacol, 0.3 ml of H_2_O_2_, and 2.5 ml of phosphate buffer and the absorbance was read at 470 nm. Change in the absorbance of 0.01 as unit per minute was defined as one unit of POD.

### Lipid peroxidation (LPO)

The activity of lipid peroxidation by T-BARS was determined in the homogenate by the method used by Iqbal and coworkers [23] and the results were expressed as TBARS per minute per milliliters of plasma. In this assay, 0.1 ml of homogenate was mixed with 0.29 ml phosphate buffer, 0.1 ml of trichloroacetic acid, and 1 ml of trichlorobarbituric acid followed by heating at 95 °C for 20 min and then shifted to an ice bath before centrifuging at 2500 rpm for 10 min. The samples were read with the help of spectrophotometer at 535 nm.

### Reactive oxygen species (ROS)

The assay of reactive oxygen species (ROS) was done according to the method of Hayashi et al. [24]. In this assay, 5 ml of H_2_O_2_ standards and the homogenate was mixed with 140 ml of sodium acetate buffer with pH 4.8 in 96-well plates and incubated at 37 °C for 5 min. After the incubation, 100 ml of DEPPD and ferrous sulphate mix samples were added in each well with a ratio of 1:25 and were incubated at 37 °C for 1 min. With an interval of 15 s for 3 min, the absorbance was read at 505 nm at microplate reader.

### Protein estimation

Determination of total protein content in tissues was done following a commercial diagnostic kit (AMEDA Labordiagnostik Laboratory, Austria) protocol. The results of protein were measured by plotting absorbance of the standard against samples. These values were expressed as milligram per gram of tissue.

### Hormonal analysis

Quantitative EIA kits were used for the measurement of testosterone (BioCheck Inc., USA Catalog No. BC-1115), luteinizing hormone (LH) (BioCheck Inc., USA Catalog No.BC-1031), and follicle-stimulating hormone (FSH) (BioCheck Inc., USA Catalog No.BC-1029) concentrations in the tissues and the assays were performed by the instructions with the kits. All the above assays were repeated with both inter- and intra-assay variations for more and precise results.

### Tissue histopathology

Testicular tissues (testis and epididymis) were fixed in formalin for 48 h, dehydrated with different grades of alcohol, and cleared with the help of xylene. The paraffin sections (5 μm) were cut and stained with hematoxylin and eosin for histology and morphometry. Transverse sections (10–20/group) of testicular tissues were examined under a Leica Microscope (New York Microscope Company) equipped with a digital camera (Canon, Japan).

For the morphometry, the images were taken at × 20 and × 40, and the results were done with Image J software. Area of different sections was calculated with the method of Jensen et al. [25]. From × 20 images, 30 pictures per animal were selected and the known area of different areas of intestinal space, epididymis tubules, and seminiferous tubules was measured by the software. The number of different cell types (spermatids, spermatogonia, and spermatocytes) and the area were calculated, and comparison of different groups with control was done.

### Statistical analysis

All parameters of data points showed normal distribution and hence were reported as mean ± SEM and difference was considered significant at *P* < 0.05. One way ANOVA followed by Dunnet’s multiple comparison tests was used for the comparison of different groups with control using Graph Pad Prism software.

## Results

### Bisphenol F in vitro effects on the testicular tissues antioxidants, ROS and testosterone secretions in the rat testis

Antioxidant enzymes, i.e., CAT, POD and SOD, oxidative stress markers, i.e., reactive oxygen species (ROS) and TBARS, were determined in the testicular tissues after 2 hours incubation with different concentrations of BPF (Table 1). There was no significant difference observed in the CAT, POD, and SOD activity in any of the BPF-treated groups as compared to the control.

**Table 1:** In vitro effect of Bisphenol F (BPF) on antioxidant enzymes and testosterone secretion in rat testis

Groups (*n* = 6/group)	Parameters					
	CAT (u/mgProtein)	POD (nmole)	SOD (u/mgprotein)	LPO (nM TBARS/min/mg Tissue)	Total ROS (U/g tissue)	Testosterone (ng/g tissue)
Control	9.53 ± 0.43	8.12 ± 0.60	10.51 ± 1.78	31.11 ± 1.81	29.00 ± 2.32	52.32 ± 2.02
BPF 1 ng/ml	8.39 ± 0.53	7.43 ± 0.79	11.99 ± 2.01	24.17 ± 1.11	35.60 ± 2.35	49.04 ± 2.45
BPF 5 ng/ml	7.94 ± 0.49	6.92 ± 1.13	12.08 ± 2.23	40.07 ± 2.56	29.60 ± 2.08	46.48 ± 1.60
BPF 25 ng/ml	7.95 ± 0.85	6.46 ± 1.28	13.66 ± 2.10	39.21 ± 2.85	38.60 ± 2.47*	44.71 ± 2.31
BPF 50 ng/ml	7.80 ± 1.29	7.06 ± 1.85	13.15 ± 0.32	46.97 ± 4.97*	39.00 ± 2.44*	48.76 ± 2.31
BPF 100 ng/ml	7.59 ± 1.04	7.86 ± 0.71	14.71 ± 0.85	46.91 ± 4.53*	41.40 ± 1.89**	47.14 ± 3.23

Values are expressed as mean ± SEM. *, **, ***Significant difference at probability value *P* < 0.05, *P* < 0.01, and *P* < 0.001 compared to control, respectively. ANOVA followed by Dunnett’s comparison test. *BPF* Bisphenol F

Reactive oxygen species and LPO are considered important oxidative stress markers. In BPF 50 ng/ml and 100 ng/ml treated groups, significant (*P* < 0.05) increases in LPO were observed as compared to the control. However, there was no significant increase observed in the low-dose-treated groups as compared to the control. Similarly, there is a dose-dependent augmentation in ROS levels in different treatment groups. In BPF 25 ng/ml and 50 ng/ml, significant (*P* < 0.05) increase in ROS was noticed, whereas in BPF 100 ng/ml, marked (*P* < 0.01) increase in ROS was examined as compared to the control group. Low doses of BPF did not induce any change in ROS level compared to the control group.

The levels of testosterone in the testis after 2 hour incubation with the treatment of different concentrations of BPF decreased but that difference was not significant as compared to control (Table 1).

### Bisphenol F different concentration effects on the body weight gain and testicular weight after sub-chronic administration

BPF exposure in male rats for 28 days did not show any significant change in the body weight of all treated groups as compared to the control. There was also no significant difference observed in the left testis and right testis of all the treated groups with BPF when compared to the control (Table 2).

**Table 2:** In vivo effect of subchronic Bisphenol F (BPF) on the different parameters

Parameter	Treatments (*n* = 7/group)					
	Control	BPF 1 mg/kg	BPF 5 mg/kg	BPF 25 mg/kg	BPF 50 mg/kg	BPF 100 mg/kg
Body weight gain (g)	35.00 ± 4.33	26.90 ± 5.23	25.10 ± 3.21	25.00 ± 4.33	29.20 ± 5.12	27.00 ± 4.33
Right Testis weight (g)	1.04 ± 0.05	1.18 ± 0.03	1.02 ± 0.03	1.14 ± 0.06	1.22 ± 0.05	1.12 ± 0.07
Left testis weight (g)	1.16 ± 0.02	1.16 ± 0.04	1.12 ± 0.02	1.14 ± 0.05	1.16 ± 0.03	1.12 ± 0.05
SOD (u/mg protein)	45.14 ± 1.19	34.29 ± 3.75	34.38 ± 1.48	36.26 ± 5.03	31.97 ± 4.63	32.54 ± 3.38
POD (nmole)	16.55 ± 0.43	15.18 ± 0.67	13.70 ± 1.12*	12.35 ± 0.39**	13.15 ± 0.64**	13.71 ± 0.68*
CAT (u/mg Protein)	16.08 ± 0.73	14.12 ± 1.01	13.03 ± 0.57*	12.99 ± 0.70*	12.91 ± 1.01*	11.59 ± 0.59**
LPO (min/mg Tissue)	13.13 ± 0.73	12.02 ± 0.80	13.35 ± 0.32	14.93 ± 0.58	15.35 ± 0.38*	15.62 ± 0.50*
Total ROS (U/g tissue)	0.94 ± 0.20	0.89 ± 0.05	0.96 ± 0.20	2.29 ± 0.42	2.78 ± 0.47*	3.11 ± 0.61**
Protein (mg/0.5 g)	341.91 ± 6.45	287.90 ± 21.72	280.23 ± 6.62*	278.90 ± 11.16*	274.77 ± 7.90*	267.81 ± 12.44**

Values are expressed as mean ± SEM. *, **, ***Significant difference at probability value P < 0.05, P < 0.01, and P < 0.001 compared to control, respectively. ANOVA followed by Dunnett’s comparison test. *SOD* superoxide dismutase, *POD* peroxidase, *CAT* catalase, *LPO* lipid peroxidation, *ROS* reactive oxygen species

### Bisphenol F different concentration sub-chronic effects on the biochemical parameters of rat testis

Antioxidant enzymes in the testicular tissues after 28 days of different concentrations of subchronic exposure to BPF and control are presented in Table 2. There was no significant difference observed in the activity of SOD when different treatment groups of BPF were compared with control. On the other hand, there was a significant difference observed in the activity of POD when different treated groups of BPF were compared with control. A significant reduction was observed in BPF 5 mg/kg (*P* < 0.05), BPF 25 mg/kg (*P* < 0.01), BPF 50 mg/kg (*P* < 0.05), and BPF 100 mg/kg (*P* < 0.05) when compared to the control.

BPF treatment caused significant (*P* < 0.05) reduction in CAT activity at doses of 5, 25, and 50 mg/kg treated groups as compared to control. Similarly, BPF treatment caused significant (*P* < 0.01) decline in CAT activity at dose levels of 100 mg/kg treated groups.

ROS and LPO level in different treatment groups are presented in Table 2. LPO which is a well-known oxidative stress marker was determined in the reproductive tissues. A significant (*P* < 0.05) increase in the LPO content was observed in BPF 50 mg/kg and BPF 100 mg/kg treated groups when compared to control. However, the other doses of BPF did not show a significant effect as compared to control.

Similarly, a significant increase in ROS level was observed in BPF 50 mg/kg (*P* < 0.05) when compared to control. Total ROS was increased significantly (*P* < 0.001) in BPF 100 mg/kg as compared to control. However, total ROS was not altered by BPF 1, 5, and 25 mg/kg groups when compared to the control.

Total protein in the testis showed a significant reduction in BPF 5 mg/kg (*P* < 0.05), BPF 25 mg/kg (*P* < 0.05), and BPF 50 mg/kg (*P* < 0.05) as compared to the control. On the other hand, BPF 100 mg/kg treatment group showed a significant reduction (*P* < 0.01) in protein levels as compared to control.

### Bisphenol F effects on the different hormones of male rats administrated with different concentrations for 28 days

Plasma testosterone, LH, FSH, and intra-testicular testosterone in the BPF different treated groups and control is presented in Table 3. Testosterone concentration was reduced significantly (*P* < 0.05) in BPF 25 mg/kg and 50 mg/kg treated groups. Similarly, BPF treatment caused a significant reduction (*P* < 0.01) at a dose level of 100 mg/kg. However, BPF in 1 and 5 mg/kg treated groups did not affect testosterone concentrations significantly.

**Table 3:** Subchronic effect of Bisphenol F (BPF) on the intra-testicular testosterone, plasma testosterone, luteinizing hormone (LH), and follicle-stimulating hormone (FSH) production in rats

Groups	Parameters			
(*n* = 7/group)	Plasm testosterone (ng/ml)	Intra-testicular testosterone (ng/g tissue)	LH (ng/ml)	FSH (IU/ml)
Control	6.03 ± 0.35	55.32 ± 1.14	1.71 ± 0.07	0.92 ± 0.01
BPF 1 mg/kg	4.48 ± 0.50	52.44 ± 2.71	1.62 ± 0.09	0.83 ± 0.03
BPF 5 mg/kg	4.34 ± 0.51	51.48 ± 2.01	1.52 ± 0.05	0.76 ± 0.10
BPF 25 mg/kg	3.99 ± 0.64 *	46.71 ± 1.87 **	1.43 ± 0.05*	0.63 ± 0.02**
BPF 50 mg/kg	3.69 ± 0.53 *	44.16 ± 1.14 ***	1.39 ± 0.07*	0.59 ± 0.02***
BPF 100 mg/kg	3.20 ± 0.25 **	45.34 ± 1.04 **	1.20 ± 0.04***	0.44 ± 0.03***

Values are expressed as mean ± SEM. *, **, ***Significant difference at probability value *P* < 0.05, *P* < 0.01, and *P* < 0.001 compared to control, respectively. ANOVA followed by Dunnett’s comparison test

Plasma LH concentrations reduced significantly in BPF 25 and 50 mg/kg (*P* < 0.05) as compared to the control. A significant reduction (*P* < 0.01) was also observed in BPF 100 mg/kg when compared to the control. On the other hand, BPF 1 and 5 mg/kg doses did not reduce plasma LH concentrations as compared to the control.

FSH reduced significantly in BPF 25 mg/kg (*P* < 0.01) as compared to the control. A significant reduction (*P* < 0.001) was also observed in BPF 50 and 100 mg/kg when compared to the control. On the other hand, BPF 1 and 5 mg/kg treatment did not reduce plasma FSH concentrations as compared to the control.

Intra-testicular testosterone in the testis after 28 days of exposure showed a significant reduction in BPF 25, 50, and 100 mg/kg (*P* < 0.01, *P* < 0.001, and *P* < 0.01, respectively) as compared to the control. Intra-testicular testosterone was not different in BPF 1 and 5 mg/kg treated groups than control (Table 3).

### Morphological changes in testes and epididymis after exposure to bisphenol F (BPF)

**Fig. 1:**
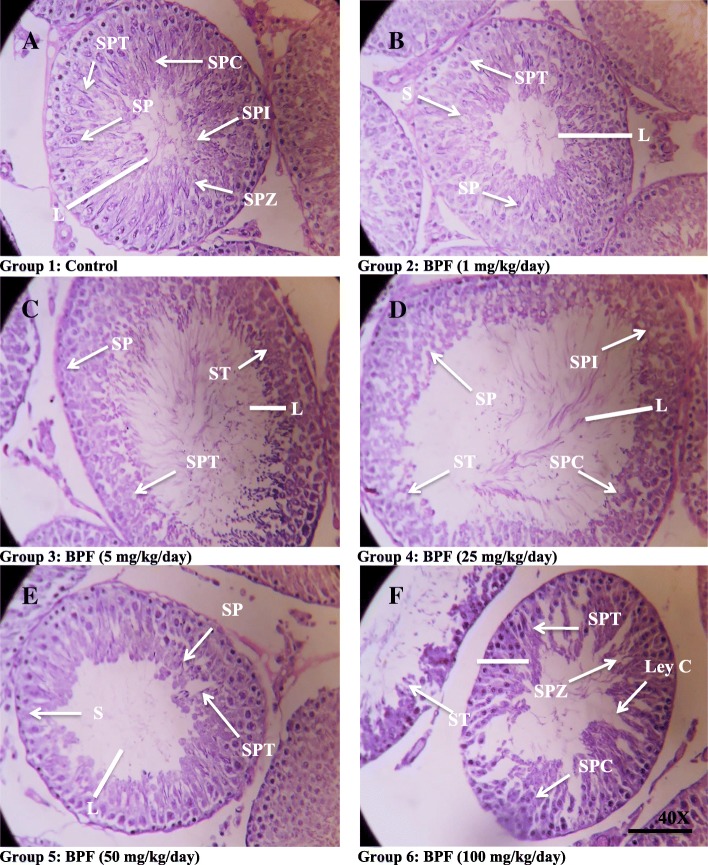
Photomicrographs of rats testicular tissues of control and treated animals with different concentrations of BPF. The control (**a**) reveals normal germ cells: spermatogonia (SP), spermatocytes (SPC), spermatids (SPT), spermatozoa (SPZ). **b**–**f** Treated groups with BPF (1, 5, 25, 50, and100 mg/kg/day) showing changes in the testicular tissues seminiferous tubules with epithelium (Line without arrowhead), showing change in the testicular parenchyma, absence of sperm in lumen, seminiferous tubules with germ cells, Leydig cells (LeyC), absence of sperm in lumen of tubules and spermatids. Presenting ST, seminiferous tubules; SP, spermatogonia; SPC, spermatocytes; SPT, spermatids; SPZ, spermatozoa; IT, interstitial tissue; LeyC, Leydig cell (White arrow). H&E (× 40)

Effect of BPF exposure on the seminiferous tubule area, interstitium area, seminiferous tubules diameter, and epithelial height in testicular tissue are presented in Table 4 and Fig. 1. There was no significant difference observed in the (%) area of seminiferous tubule and (%) area of interstitium of different treatment groups of BPF as compared to control. Similarly, a non-significant difference was observed in the diameter of seminiferous tubules in all treated groups as compared to control. There was significant (*P* < 0.0.5) reduction in epithelial height in BPF 50 mg/kg and 100 mg/kg groups when compared to the control. On the other hand, epithelial height was not different in BPF 1, 5, and 25 mg/kg treated groups than the control.

**Table 4:** Oral subchronically administered rats with Bisphenol F (BPF) testis, caput, and cauda epididymis morphometry after 28 days of exposure

Parameter		Treatments (*n* = 7/group)					
		Control	BPF 1 mg/kg	BPF 5 mg/kg	BPF 25 mg/kg	BPF 50 mg/kg	BPF 100 mg/kg
Testis	Area of seminiferous tubule (%)	90.02 ± 0.98	88.85 ± 2.18	88.87 ± 2.34	87.37 ± 1.34	86.73 ± 1.41	85.65 ± 2.56
	Area of Interstitium (%)	19.02 ± 0.79	18.20 ± 0.88	17.43 ± 0.37	17.27 ± 0.54	16.88 ± 0.41	15.90 ± 1.50
	Seminiferous tubule diameter (μm)	213.91 ± 2.51	211.08 ± 5.49	209.09 ± 2.81	208.98 ± 0.72	207.48 ± 0.84	207.47 ± 1.48
	Epithelial height	77.27 ± 1.94	74.47 ± 1.95	73.71 ± 3.02	72.38 ± 1.40	69.16 ± 1.30*	68.13 ± 2.07*
Caput	Tubular diameter (μm)	403.40 ± 6.76	399.60 ± 4.28	398.20 ± 3.78	396.20 ± 3.94	394.80 ± 4.61	396.40 ± 2.97
	Lumen diameter (μm)	300.00 ± 7.05	298.60 ± 7.50	295.00 ± 4.14	297.20 ± 4.57	292.60 ± 4.82	290.60 ± 4.34
	Epithelial height (μm)	31.40 ± 1.80	29.40 ± 3.84	28.00 ± 1.41	28.20 ± 3.10	27.00 ± 0.83	26.60 ± 1.16
	Epithelium (% age)	37.95 ± 1.99	34.20 ± 1.01	33.60 ± 0.81	32.80 ± 1.49	32.40 ± 2.59	32.00 ± 0.63
	Lumen (% age)	70.85 ± 1.65	69.00 ± 3.21	67.86 ± 1.78	67.25 ± 0.91	68.34 ± 2.40	66.25 ± 7.74
Cauda	Tubular diameter (μm)	482.80 ± 4.58	478.60 ± 3.60	476.60 ± 5.26	477.80 ± 4.03	475.80 ± 6.51	474.80 ± 4.03
	Lumen diameter (μm)	432.60 ± 2.98	436.80 ± 4.68	435.20 ± 4.65	441.00 ± 4.32	438.80 ± 0.96	439.20 ± 3.36
	Epithelial height (μm)	33.25 ± 2.32	34.50 ± 1.79	35.75 ± 0.49	35.80 ± 4.03	36.20 ± 2.07	38.40 ± 3.40
	Epithelium (% age)	39.00 ± 1.54	43.75 ± 1.89	44.75 ± 2.10	42.75 ± 2.41	44.10 ± 1.73	45.00 ± 1.18
	Lumen (% age)	63.25 ± 1.45	60.00 ± 2.87	62.25 ± 1.73	60.50 ± 2.77	59.25 ± 3.04	58.65 ± 2.47

Values are expressed as mean ± SEM. *Significant difference at probability value *P* < 0.05 compared to control. ANOVA followed by Dunnett’s comparison test

Transverse sections of testicular tissues of the control group were observed with thick epithelium, sperm-filled lumen, and seminiferous tubules (Fig. 1). Seminiferous tubule arrangement and shape was not very different in all treated groups when compared to the control. Though the pattern of epithelium was thin and the number of secondary spermatocytes was reduced in the treated groups when compared to the control. However, the groups with the higher doses of BPF were observed with few tubules and there were very few elongated spermatids in the lumen when these groups were compared with the control (Fig. 1).

**Fig. 2:**
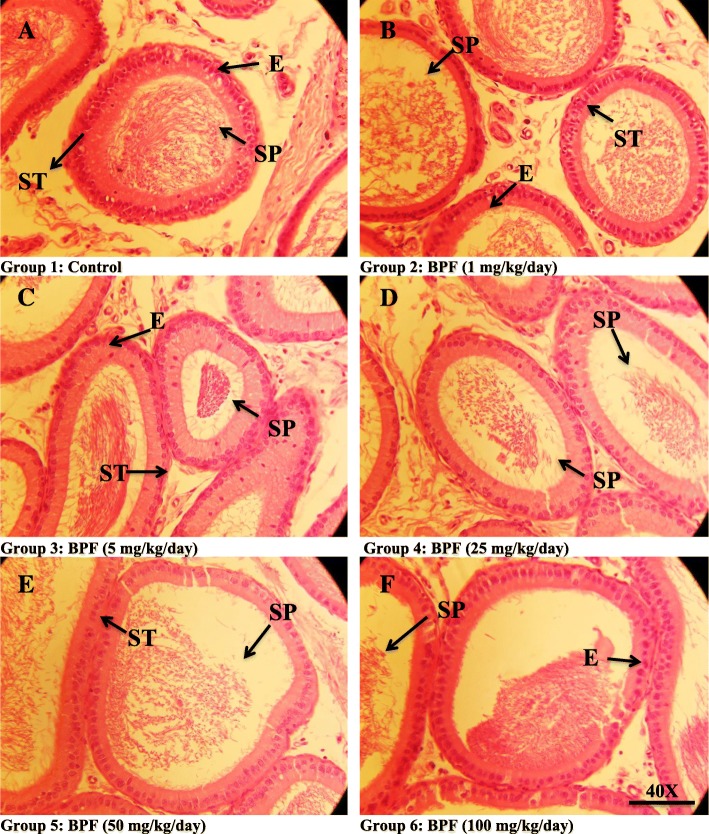
Photomicrograph of caput epididymis tissue showing **a** control; with compact arrangement of caput tubules with sperm-filled lumen **b** BPF (1 mg/kg/day)-exposed group, presenting normal caput tubules like in the control. **c** BPF (5 mg/kg/day) exposed group showing seminiferous tubules with less number of sperm in the lumen (arrow). **d** BPF (25 mg/kg/day)-exposed group presenting caput tubules with empty lumen (arrow). Similarly, **e** BPF (50 mg/kg/day)-exposed group showing less number of sperms in the lumen. **f** BPF (100 mg/kg/day)-exposed group showing less number of sperms and empty lumen (arrow). Presenting SP, spermatozoa; ST, seminiferous tubules; E, epithelium. H&E (× 40)

Morphometry of different parameters of caput and cauda epididymis region after different BPA exposures did not show any significant difference in any of the parameter (tubular and lumen diameter, epithelial height, and percentage of epithelium and lumen) as compared to the control presented in Table 4 and Fig. 2. The shape of cauda and caput of the epididymis in the control was not very different from that of the treated groups. In the groups treated with 25, 50, and 100 mg/kg/day there were few empty lumens observed in each epididymis section when compared to the control though there was no loss of stereocilia observed.

**Fig. 3:**
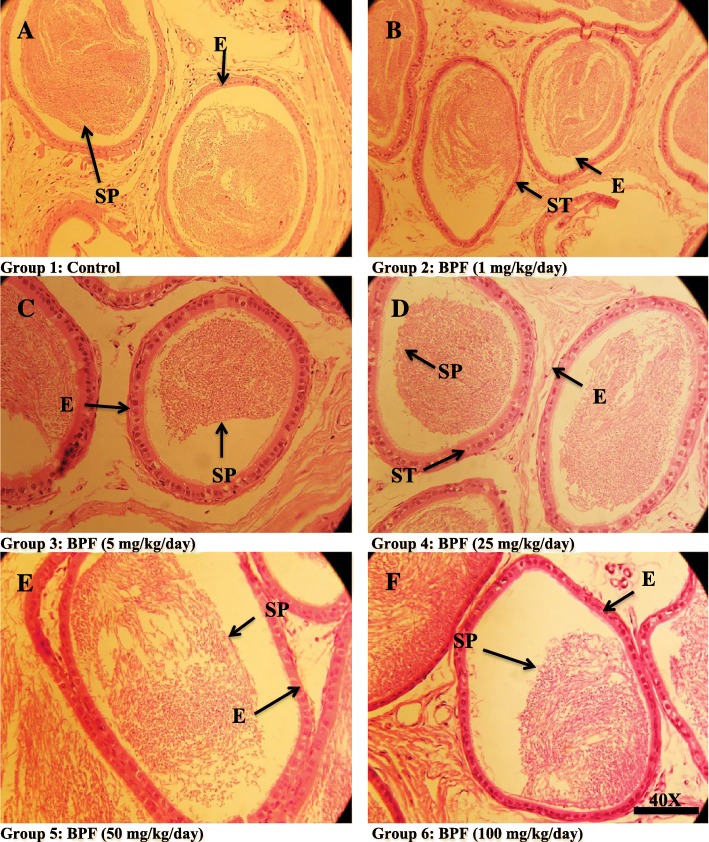
Photomicrograph of cauda epididymis tissue showing **a** control; with compact arrangement of cauda tubules with sperm-filled lumen. **b** BPF (1 mg/kg/day)-exposed group, presenting normal caput tubules like in the control. **c** BPF (5 mg/kg/day)-exposed group, presenting cauda tubules with sperm-filled lumen. **d** BPF (25 mg/kg/day-exposed group presenting cauda tubules with less sperm in the lumen. Similarly, **e** BPF (50 mg/kg/day)-exposed group presenting cauda tubules with fewer sperm in the lumen. Likewise, **f** BPF (100 mg/kg/day)-exposed group presenting cauda tubules with empty spaces and fewer number of sperm in the lumen. Presenting SP, spermatozoa, ST, seminiferous tubules; E, epithelium. H&E (× 40)

The number of different cell types in the seminiferous tubules presented in Fig. 3. A significant difference was not observed in any of the treated group with different concentrations of BPF as compared to the control. Though the number of cells like spermatids and spermatocytes had decreased in some of the treated groups when compared to the control, the reduction was not statistically different when the comparison was done with the control.

## Discussion

Although numerous studies have been published on the effects of BPA on the reproductive functions of male rats, the underlying mechanisms remain unclear. BPA displays non-monotonic dose-response functions [26]. Current knowledge on the biological and potential toxicological effects of BPA analog, especially on the reproductive system, is limited. The main purpose of the current study was to understand how safe is the BPA analog “BPF” from the medical point of view using rat models. Increased exposure of BPA during the pre-pubertal and pubertal period may affect the normal development and functions of reproductive organs, and the resulting toxic effects of these chemicals may affect the regulatory genes involved in the development of follicles in females and sperms in males. The consumption of BPA alternatives is at rising due to strict regulations on the use of BPA in some countries [27, 28]. A study suggested a possible association between BPA levels and increased risk of prosocial behavior and between MECPP levels and increased risk of conduct problems [29]. The structural similarity of BPF with BPA marks it as an endocrine disruptor and in vitro data has also revealed that BPF has a binding affinity with receptors which change the testosterone secretions in the fetal testis and can also induce cell proliferation. In vivo studies have shown that BPF influences the expression of sex hormone-regulated genes and also has developmental and reproductive effects in mammals. Recently published data regarding some of the BPA analogs has upturned concerns that whether the so-called safer analogs of BPA are more alarming to both human and wildlife [30]. In the present study, we conducted both in vivo and in vitro studies to evaluate the effects of BPF on the reproductive functions of male rats.

In the in vitro study, we incubated testicular tissues with different concentrations of BPF for 2 hours. The incubated tissues did not show any significant change on the antioxidant enzymes as CAT, SOD, and POD. BPA and its analogs have a toxic influence on the formation of ROS inside the body, and studies have also shown that these phenols not only increase in the levels of ROS but also lead into oxidative stress inside many cellular networks [31]. Similar to data reported in BPS, we observed increase ROS and lipid peroxidation in testicular tissues after exposure to BPF in vitro [16, 32]. The results of the in vivo study showed dose-dependent effects of BPF on the oxidative stress in the reproductive system of male rats. The groups exposed to higher concentrations of BPF showed a significant difference in the histology of the reproductive tissues by reducing the sperm number in the epididymis and decreasing the height of epithelial tissues. Androgens also play an important role in the normal development of the male reproductive system [26, 33, 34]. BPF higher exposure groups were observed with an elevated level of testosterone which also leads to higher oxidative stress as compared to the low-dose-exposure groups.

Exposure to BPF caused induction of ROS which lead into an increase in the levels of LPO and activation of antioxidant enzymes which are in line with the earlier studies where BPA exposure degraded protein and altered antioxidant enzymes [35]. In the in vivo study, BPF exposure also increased the levels of LPO and also altered the levels of SOD and CAT which also indicated oxidative stress. This change occurred because of oxidative stress in the reproductive tissues caused by exposure of BPF different concentrations which also reduced the levels of proteins and antioxidant enzymes which are similar to the findings of some previous studies [36–39].

We observed the substantial change in both plasma and intra-testicular testosterone in the in vivo study. Our results are in accordance with previous researches which indicated altered levels of different hormones after exposure to BPA and some of its analogs [19, 34].

The process of Spermatogenesis is controlled by different reproductive hormones and cellular interactions inside the testes. ROS and disturbed antioxidant enzymes lead to disturbed spermatogenesis [40]. In the testicular tissues, we observed a reduction in the number of spermatids, alerted epithelial height and seminiferous tubules, and reduced concentrations of testosterone. Some previous studies are in accordance with our current study on the exposure of BPF where exposure to BPA and some of its analogs altered steroidogenesis and lead into oxidative stress in the different tissues [19, 34]. Similarly, our current study results also showed that BPF not only alters spermatogenesis in the testis but also causes a reduction in the levels of testosterone secretions. Further studies are required both in vitro and in vivo which can show the molecular and cellular mechanisms of these BPA analogs specific response in the environmental hazard assessment which will let us better understand the mechanisms through which BPA analogs endocrine disruption on different tissues be analyzed.

## Conclusions

The results of our present study showed that BPF at higher dose exposures may possibly have outcomes in oxidative stress and disturbed reproductive hormones. Thus, the use of BPA analogs should be carried out with caution, especially until the effective risk assessment is conducted. Further studies need to analyze the molecular basis of these alterations both in vivo and in vitro studies which will let us understand how BPF can still have an effect on the physiology of different tissues inside the body.

## Data Availability

Not applicable
